# Genomic deletions in *Aureobasidium pullulans* by an AMA1 plasmid for gRNA and CRISPR/Cas9 expression

**DOI:** 10.1186/s40694-024-00175-4

**Published:** 2024-06-01

**Authors:** Audrey Masi, Klara Wögerbauer, Robert L. Mach, Astrid R. Mach-Aigner

**Affiliations:** 1 Christian Doppler Laboratory for Optimised Expression of Carbohydrate-Active Enzymes, Institute of Chemical, Environmental and Bioscience Engineering, TU Wien, Gumpendorfer Str. 1a, A-1060 Vienna, Austria; 2 Institute of Chemical, Environmental and Bioscience Engineering, TU Wien, Gumpendorfer Str. 1a, A-1060 Vienna, Austria

**Keywords:** Fungal transformation, Protoplasts, *Aureobasidium pullulans*, CRISPR/Cas9, Golden Gate Assembly, AMA1 plasmid

## Abstract

**Background:**

*Aureobasidium pullulans* is a generalist polyextremotolerant black yeast fungus. It tolerates temperatures below 0 °C or salt concentrations up to 18%, among other stresses. *A. pullulans* genome sequencing revealed a high potential for producing bioactive metabolites. Only few molecular tools exist to edit the genome of *A. pullulans*, hence it is important to make full use of its potential. Two CRISPR/Cas9 methods have been proposed for the protoplast-based transformation of *A. pullulans*. These methods require the integration of a marker gene into the locus of the gene to be deleted, when the deletion of this gene does not yield a selectable phenotype. We present the adaptation of a plasmid-based CRISPR/Cas9 system developed in *Aspergillus niger* for *A. pullulans* to create deletion strains.

**Results:**

The *A. niger* CRISPR/Cas9 plasmid led to efficient genomic deletions in *A. pullulans.* In this study, strains with deletions ranging from 30 to 862 bp were obtained by using an AMA1 plasmid-based genome editing strategy.

**Conclusion:**

The CRISPR/Cas9 transformation system presented in this study provides new opportunities for strain engineering of *A. pullulans.* This system allows expression of Cas9 and antibiotic resistance while being easy to adapt. This strategy could open the path to intensive genomic engineering in *A. pullulans*.

**Supplementary Information:**

The online version contains supplementary material available at 10.1186/s40694-024-00175-4.

## Background

### *Aureobasidium pullulans*: an industrial producer with great potential

*Aureobasidium pullulans* is an ascomycete from the Dothioraceae family. It is an ubiquitous black-yeast-like fungus that can survive many stresses such as high salinity, extreme pH condition or extreme temperature and, is therefore found in a diversity of habitats from hypersaline water to polar environment [1–3]. *A. pullulans* is renowned for pullulan production, a water-soluble exopolysaccharide [4, 5]. Widely used in the food and pharmaceutical industry, pullulan has an estimated market between USD 68 million and 130 million, depending on the source [6, 7]. *A. pullulans* also produces several secondary metabolites of interest, encompassing siderophores, β-L malic acid, liamocin oil, and has a capacity to secrete many proteins such as lipases and amylases [8–13]. As a biocontrol agent, *A. pullulans* demonstrated efficacy against post-harvest molds, such as *Botrytis cinerea, Colletotrichum acutatum and Penicillium* spp. [14–16]. The mechanisms underlying *A. pullulans* efficacy as biocontrol agent are not fully understood [14–17]. It may be explained by its capacity to secrete extracellular lytic enzymes and antifungal compounds, such as exophilins, liamocins and free fatty acid [14–18]. The sequencing and analysis of *A. pullulans* genomes have allowed the prediction of biocontrol genes or secondary metabolites [2, 3, 18]. However, gene function needs—besides genomic prediction—confirmation by targeted mutation/deletion using genome editing. Unfortunately, the limited *A. pullulans* engineering toolbox is slowing the pace of such experimental work. *A. pullulans* has a remarkable phenotypic plasticity, growing either yeast-like or filamentous. This suggests the possibility of using engineering toolboxes from yeasts and/or filamentous fungi.

### Available tools for *A. pullulans* genome editing

The current standard method for transforming *A. pullulans* is PEG-mediated protoplast transformation. Two research groups described CRISPR/Cas9-mediated genome editing in *A. pullulans* [19, 20]. Zhang et al. co-transformed two plasmids with yeast promoters and terminators: one for Cas9 expression and another for expressing a gRNA targeting the *URA3* gene [20]. In yeasts, the *URA*3 gene (systematic name YEL021W) encodes the protein orotidine 5’-phosphate decarboxylase, which catalyses the formation of uridine monophosphate. *URA*3 deficient strains are auxotrophic and require supplementation with uridine for growth. They are also resistant to 5-fluoroorotic acid (5-FOA), an otherwise toxic compound due to its metabolization into fluorouracil. Therefore, the deletion of the *URA3* gene creates a selection marker. Orthologous genes of *URA*3 in filamentous fungi are *pyr*G in *Aspergillus* sp and *pyr*4 in *Trichoderma* sp., which are also used as marker genes. However, the work of Zhang and colleagues was the first report on efficient CRISPR/Cas9-mediated genome editing in *A. pullulans*. They demonstrated that this method led to the efficient deletion of the *URA3* gene and that CRISPR/Cas9 improved the efficiency of homologous recombination [20].

Kreuter et al. used a ribonucleoprotein (RNP) approach, delivering the Cas9 enzyme and gRNAs in the cell during transformation [19]. They also successfully disrupted *URA3* and improved homologous recombination efficiency [19].

### Drawbacks of current CRISPR/Cas9 approaches

Deleting the *URA3* gene creates a selectable phenotype as it confers the recombinant strain resistance to 5-FOA. However, many gene deletions do not lead to selectable phenotypes. In such cases, a gene conferring an antibiotic resistance needs to replace or disrupt the gene targeted for deletion. Alternatively, an auxotrophic strain is used as parental strain for the deletion and prototrophy is restored during the transformation by inserting the missing gene in the locus targeted for deletion. Both options have their disadvantages. Antibiotic resistance markers should be avoided for industrial applications. Their presence complicates the acceptance by the authorities of the strain and the manufacturing process using it [21, 22]. It also involves a risk of carry-over the resistance marker DNA into the final product, which would represent a safety hazard and requires additional controls in certain industries [23, 24]. Generation of auxotrophic strains is cumbersome. It requires to identify a suitable auxotrophic marker, deleting this gene (using another marker eventually, if the deletion does not create a selectable phenotype). For some fungal strains, the catalogue of available markers is limited, thus generating multiple deletion strains with this strategy requires to recycle the markers. The above-mentioned CRISPR/Cas9 approaches require either an antibiotic marker or an auxotrophic strain for deleting a gene not linked to a selectable phenotype. In *Aspergillus niger,* a solution for this challenge has been found by using AMA1-based plasmids for CRISPR/Cas9-based genome editing.

### AMA1-derived plasmids

AMA1, Autonomously Maintained in *Aspergillus*, is a DNA sequence with an inverted repeat promoting extrachromosomal replication [25–27]. AMA1 was initially identified in a plasmid isolated from *A. nidulans* and was later demonstrated to allow plasmid replication also in *A. niger* [25–27]. Sarkari et al. used this property of the AMA1 sequence to construct a Golden Gate assembly (GGA) system with a self-replicating CRISPR/Cas9 plasmid for metabolic engineering in *A. niger* [28]. The AMA1 sequence used in this self- replicating CRISPR/Cas9 plasmid was shortened by about half (5.3–2.8 kb). Sarkari et al. tested different plasmids with various sizes of the AMA1 fragment and demonstrated that the half shorten AMA1 fragment was the best compromise tested to maintain efficient transformation in *A. niger* while promoting loss of the plasmid under no selection pressure and the possibility to replicate in *E. coli* [28]. The final version of the created CRISPR/Cas9 self-replicating plasmid (P2 in supplementary Table 1) contained a gRNA under the control of the *mbfA* promoter from *A. niger*, a Cas9 encoding sequence expressed under the *coxA* promoter from *A. niger*, the shortened AMA1 sequence and an hygromycin B resistance cassette from the vector pRLM_Ex30_ (hygromycin B resistance under the control of the *pki* promoter from *T. reesei)* [28]. They demonstrated that this plasmid generates deletion mutations in the genomic DNA at the locus targeted by the gRNA and allows selection with hygromycin B without causing genomic integration of the hygromycin B resistance encoding marker or Cas9 coding sequence.

### Novelty of this study

The method presented in this study uses the GGA system and the CRISPR/Cas9 constructed by Sarkari et al. for *A. niger* (P2 in supplementary Table 1). We demonstrate that this system is efficient in *A. pullulans* to obtain deletion strains. This work is the first report of a CRISPR/Cas9 transformation system using only a single plasmid in *A. pullulans*. This system avoids prior auxotrophic strain construction and is easily adaptable for new targets.

## Materials and methods

### Strains and cultivation conditions

*A. pullulans* EXF-150 (CBS 100280) was used as the wild-type and a parent strain for disrupting the *URA3* gene [3]. *A. pullulans* was cultivated at 24 °C on malt extract (MEX) agar plates (1 g/L peptone, 30 g/L malt extract, 15 g agar in tap water). For selection, a minimal medium described by Ueda et al. was used: 0.06% (NH_4_)_2_SO_4_, 0.1% NaCl, 0.5% K_2_HPO_4_, 0.02% MgSO_4_∙7H_2_O, 0.4% yeast extract and 2% carbon source (added after autoclaving) [29]. The salts were prepared in a fivefold concentrated stock at pH 6 (adjusted with HCl). Uridine at a final concentration of 5 mM was added to the medium to cultivate the uridine auxotrophic strain. To select for 5-FOA or hygromycin B resistance, these compounds were added at the respective final concentrations of 2 g/L or 100 μg/mL.

### Construction of the CRISPR/Cas9 plasmid (adapted from [28])

**Fig. 1:**
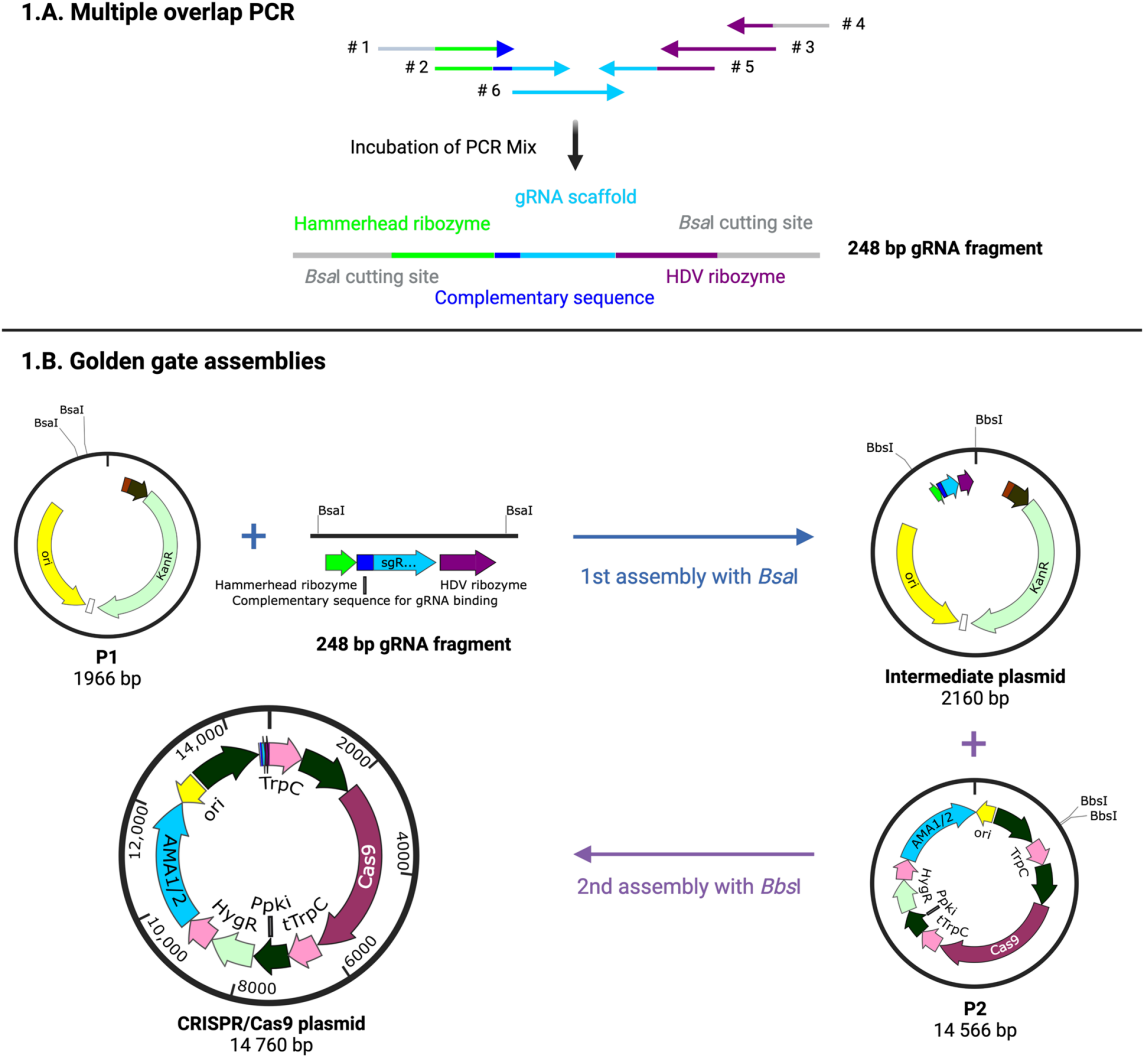
Overview on the CRISPR/Cas9 plasmid assembly. **A**. Scheme of the multiple overlap PCR to generate the 248 bp fragment. This fragment was assembled by PCR using two primers containing the sequence specific to the gRNA target and four generic primers. The obtained product includes a hammerhead ribozyme, the complementary sequence to the target DNA, the sequence for expression of the gRNA, a hepatitis delta virus (HDV) ribozyme for cutting at the 5’ and 3’ ends of the gRNA and *Bsa*I restriction sites on the 3’ and 5’ ends. **B**. Overview on the two GGAs to generate the final CRISPR/Cas9 plasmid. The initial GGA using *Bsa*I*,* leads to the ligation of the 248 bp-fragment and the BB1_L_23_syn_BsaI plasmid. The product of this assembly is then assembled with the plasmid bearing the Cas9 expression cassette via *Bsb*I

We aimed for deletion of the gene coding for the orotidine-5-phosphate carboxylase (gene ID: 40743721) using a gRNA target described by Kreuter et al. [19] (no. 14 in Supplementary Table 1). The CRISPR/Cas9 plasmid was assembled in three steps, as displayed in Fig. 1. First, a 248 bp-fragment containing the gRNA was created by multiple overlap extension PCR (MOE-PCR): six primers with overlaps (oligonucleotides no. 1–6 in Supplementary Table 1) were used to generate the fragment in one PCR (Fig. 1A). The 50 µL PCR mix contained 20 µM dNTPs, 0.5 µM of each gRNA primer, 0.25 µM of each structural primer, 1U Q5 DNA polymerase, and Q5 buffer. The reaction was incubated in a thermocycler for 1 min 30 s at 95 °C followed by 35 cycles of 30 s at 95 °C, 20 s at 60 °C and 10 s at 72 °C and followed by the final extension for 5 min at 20 °C. The generated PCR product was analyzed on an agarose gel, and the correct band was cut out and purified [28].

Then, this 248 bp-fragment was assembled with the plasmid P1 (Supplementary Table 1) through a GGA with *Bsa*I (Fig. 1B)*.* The resulting plasmid was assembled with the plasmid P2 (Supplementary Table 1) using GGA with *Bbs*I (Fig. 1B). GGA were performed in a final volume of 20 µL containing 2 mM of each construct to assemble, CutSmart Buffer, 100 U of T4 ligase, 2 mM ATP, 30 U *Bsa*I-HF or *Bbs*I (New England Biolabs). The assemblies were incubated in a thermocycler for 45 cycles of 2 min at 37 °C and 5 min at 16 °C followed by a single step of 5 min at 50 °C and 10 min at 80 °C. The resulting products were transformed into *E. coli* Top10 and selected on LB agar containing the relevant antibiotic.

### Protoplast generation (adapted from [19])

For the generation of protoplasts, a cell suspension of *A. pullulans* in a sterile solution of 0.8% NaCl/0.05% Tween 80 was prepared by scraping cells from an agar plate. A shake flask containing 20 mL MEX medium was inoculated with the suspension with a target starting OD of 0.05 and incubated overnight (~ 16 h) at 220 rpm and 24 °C. 20 mL of the culture (OD_600_ = 1–2) was centrifuged at 4000 × g for 5 min at room temperature (RT). The supernatant was discarded, and the pellet was resuspended in 20 mL of buffer A (100 mM KH_2_PO_4_, 1.2 M sorbitol, pH = 5.6, sterilized by autoclaving). The suspension was centrifuged at 4000 × g for 5 min at RT, then the supernatant was removed, and the pellet was resuspended in 15 mL sterile lysing solution (600 mg VinoTaste^®^ Pro lysing enzymes (Novozymes, catalogue number 2860–689-03–2) dissolved in 15 mL buffer A, sterile filtered). The suspension was placed in a shake flask and incubated in a rotary shaker at 140 rpm and 24 °C for 45–60 min until protoplasts were formed. The protoplast suspension was poured into a 50 mL reaction tube, filled with up to 40 mL with ice-cold 1.2 M sorbitol, and centrifuged at 3000 × g for 10 min at 4 °C. The formed pellet was washed once with 30 mL 1.2 M ice-cold sorbitol and twice with 10 mL ice-cold, sterile buffer B (1 M sorbitol, 10 mM Tris–HCl (pH = 7.5), 25 mM CaCl_2_, sterilized by autoclaving), each time centrifuged at 3000 × g for 10 min at 4 °C. The protoplasts were handled gently and always kept on ice. The protoplast suspension and 20% PEG solution (1/3 sterile 60% PEG solution, 2/3 sterile buffer B) were mixed 1:1, and DMSO was added to a final concentration of 1% and mixed carefully. The 60% PEG solution was made of 600 g/L PEG 4000, 10 mM Tris pH = 7.5 and 10 mM CaCl_2_. Protoplasts were counted with a Thoma cell counting chamber to verify that the concentration is approximately 10^6^–10^7^ protoplasts per mL. The protoplast suspension was aliquoted (200 µL aliquots) in prechilled reaction tubes and stored on ice (for protoplasts used freshly) or at − 80 °C (for frozen protoplasts). Two transformations were performed for which two different batches of protoplasts were used. The first transformation was made with frozen protoplasts only, and the second transformation was made with fresh and frozen protoplasts from the same batch [19].

### Transformation of protoplasts (adapted from [19])

A 200 µL-aliquot of protoplasts, either freshly prepared or frozen and thawed on ice, was used. The aliquot was mixed with 2 or 5 μg undigested plasmid, 2 μL β-mercaptoethanol and 50 μL buffer B. The mix was incubated on ice for 30 min. 50 µL, 200 μL and 500 μL 60% PEG solution were added stepwise to the mix, followed by careful mixing between each step. The mix was incubated at RT for 20 min. Then, 200 μL, 400 μL, 1000 μL and 2500 μL of sterile buffer C (1 M sorbitol, 10 mM Tris–HCl (pH = 7.5)) were added stepwise with careful mixing after each step. The final volume of the transformation solution was 5.2 mL. 20 mL of melted selection medium containing 1 M sucrose and 100 μg/mL hygromycin B was added to the protoplast suspension and poured into plates. Different volumes of the protoplast suspensions were used. The plates were incubated in the dark at 24 °C until colonies were visible (4–14 days) [19].

### Selection of transformants

Hygromycin B-resistant transformants were streaked on plates containing 5-FOA. Some positive transformants on 5-FOA were randomly selected and underwent two isolation passages on MEX plates supplemented with uridine. Transformants were considered resistant to 5-FOA when colonies grew better than the negative control (wild-type strain EXF-150) and similarly to the positive control (strain #6 (∆URA3 mutant, generated with the RNP method [19])). The colony forming units per µg DNA (CFU) were estimated as follows:

1 $$\frac{Number\, of\, transformants\, on\, hygromycin }{{Quantity\, of\, DNA\, used\, for\, transformation}}*\left( {\frac{Total\, volume\, of \,protoplasts\, solution}{{Volume\, of\, protoplasts\, solution\, used}}} \right)$$

### Colony PCR and sequencing

After the first transformation assay, 2 transformants resistant to 5-FOA and hygromycin B were selected for colony PCR with primers 7 and 8 (Supplementary Table 1). The colony PCR was performed following the protocol of Wu et al. for rapid screening of yeast colonies [30]. 2 μL of the fungal cell suspension was used as a template in a 50 μL colony PCR using Q5 DNA polymerase (NEB) and the wild-type strain as a control. The molecular mass of the PCR products was checked on an 0.8% agarose gel. For visualization, a Bio-Rad ChemiDoc Imaging System was used. PCR products of the correct molecular mass were purified with the Thermo Scientific GeneJET PCR Purification Kit and sent for sequencing to Microsynth AG with the same primers as for PCR, primers 7 and 8.

### Extraction of genomic DNA, PCR and sequencing

After the second transformation assay, no colony PCR was performed. Isolated transformants were directly used for DNA extraction and PCR on the genomic material. DNA extraction was performed on 33 transformants from the second transformation assay and one transformant from the first transformation assay. The selected transformants were cultivated overnight in liquid MEX medium, then centrifuged and about 50 mg of the cell biomass was used as starting material for the DNA extraction. 50 mg of cells were placed in a 2 mL tube with glass beads (0.37 g of 0.1 mm diameter beads, 0.25 g diameter of 1 mm diameter beads and one bead of 5 mm diameter) in 1 mL CTAB buffer (1.4 M NaCl, 100 mM TrisCl pH 8.0, 10 mM EDTA, 2% CTAB and 1% polyvinylpyrrolidone) and mechanically disrupted using a FastPrep high-speed homogenizer (MP Biomedicals) at 4 m/s for 30 s. The tube was then incubated for 20 min at 65 °C. The supernatant and foam were transferred to a 2 mL tube and mixed with 400 μL phenol and 400 μL chloroform by manual horizontal shaking. Then an incubation step of 5–10 min at room temperature was performed followed by centrifugation at 12,000 g at 4 °C for 10 min. After centrifugation, 650 μL of the top phase were transferred to a 1.5 mL tube, 650 μL chloroform were added and the tube mixed by manual horizontal shaking for 30 s, followed by centrifugation at 12000 g at 4 °C for 10 min. 500 μL of the top phase were transferred to a new 1.5 mL tube and 4 μL of RNAse A at 10 mg/mL added to the tube, the tube was mixed by inversion 6 times and incubated 30 min at 37 °C. After incubation 350 μL of isopropanol were added to the tube, the tube was mixed by inversion 6 times and incubated 10 min at room temperature. Then DNA was precipitated by centrifugation at 21000 g 4 °C for 30 min. The supernatant was removed and 1 mL of ice cold 70% ethanol added, followed by centrifugation at 21000 g 4 °C for 10 min. The supernatant was removed and the remaining ethanol evaporated by incubation of the tube at 50 °C. The obtained pellet was dissolved in 50 μL ultrapure water or TE buffer. The concentration and purity were checked using NanoDropOne^C^ (Thermo Fisher Scientific) and agarose gel electrophoresis. A PCR was performed using Q5 DNA polymerase (NEB) according to manufacturer protocol with the primers 9 and 10 or 11 and 12 (Supplementary Table 1). Sequencing was performed by Microsynth AG using primers 10, 11 and 12 (Supplementary Table 1).

### Phenotype analysis

After one passage on plates with uridine and 5-FOA and two isolation passages on MEX plates, the transformants selected for DNA extraction were grown on MEX plates supplemented with uridine, MEX plates supplemented with uridine and hygromycin B and MEX plates supplemented with uridine and 5-FOA. The wild-type and a mutant bearing a genomic hygromycin B resistance were used as controls. Plates were incubated at 25 °C for 2–3 days.

## Results

### Success of transformations

Two transformations were performed to test the efficiency of transformation in *A. pullulans* with the single CRISPR/Cas9 plasmid generated in this study*.* The goal of the first assay was to test if the system would work at all, while the goal of the second transformation was to compare more in detail the effect of the quantity of DNA used in the transformation, the status of the protoplasts (fresh or frozen) and whether obtained results observed in the first transformation are reproducible. Table 1 summarizes the results of the two transformations performed. It provides the number of transformants obtained depending on the CRISPR/Cas9 plasmid quantity used. In the first transformation, performed with frozen protoplasts, 150 transformants were obtained on a hygromycin B selection plate with 2 µg plasmid, and 250 were obtained with 5 µg plasmid. Out of the 126 selected hygromycin B-resistant transformants, 105 (83%) were also resistant to 5-FOA. In the second transformation, fresh and frozen protoplasts were transformed. The obtained protoplast suspensions were divided into different volumes mixed with melted agar and poured in Petri dishes. Out of the 33 selected hygromycin B-resistant transformants, all were also resistant to 5-FOA. The transformation with the CRISPR/Cas9 was efficient at producing mutants with the expected phenotype. Increasing the quantity of plasmid DNA used increased the amount of transformants obtained, whereas the effect on the efficiency of the transformation (CFU per µg DNA used) is unclear. The use of fresh protoplasts led to more transformants and higher efficiency, and—despite lower efficiency—transformation with frozen protoplasts remained sufficing.

**Table 1:** Number of transformants obtained on indicated selection media

Transformation^1^	Plasmid^2^ (µg)	V proto^3^ (mL)	Hygromycin B colonies^4^ (CFU in colonies/µg DNA)^5^		5-FOA^8^
			Fresh^6^	Frozen^7^	
1	2	5.2	NT	150 (75)	105 out of 126 (83%)
1	5	5.2	NT	250 (50)	
2	2	0.2	0	0	33 out of 33 (100%)
2	2	0.5	10 (52)	5 (26)	
2	2	4.5	290 (168)	37 (21)	
2	5	0.2	0	0	
2	5	0.5	47 (98)	7 (15)	
2	5	4.5	572 (132)	116 (27)	

*NT* Not tested

^1^Number of the transformation

^2^Quantity of plasmid DNA added during the transformation

^3^Volume of the used protoplast solution plated with agar (the total volume of the solution is 5.2 mL)

^4^Number of colonies on the hygromycin B selection plate and

^5^CFU in colonies per µg DNA given in brackets and calculated based on Eq. 1

^6^The protoplasts used for the transformation were freshly prepared

^7^The protoplasts used were thawed

^8^Number of colonies on the 5-FOA selection plate after transfer from the hygromycin B plate. The results are expressed in number of colonies growing out of the total number of colonies transferred, and the representative percentage is also given

### Determination of the genomic modification

**Fig. 2:**
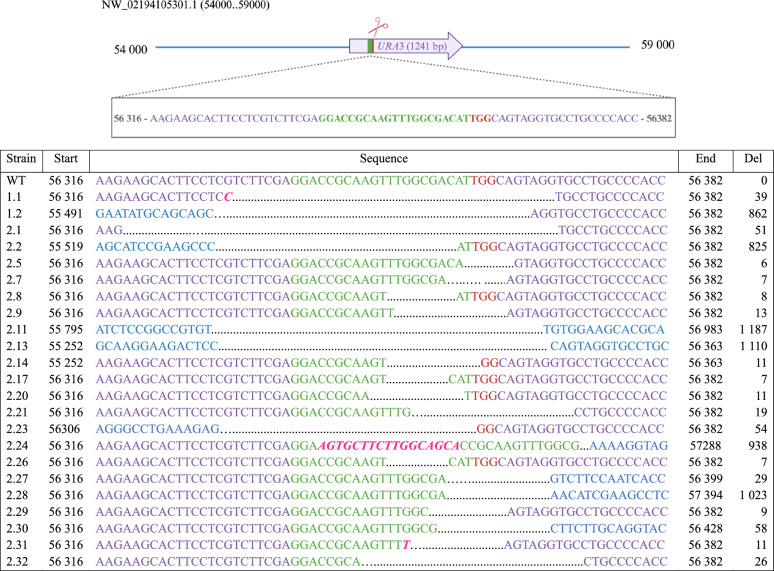
Sequencing results of obtained transformants. A schematic drawing of the *URA*3 locus is given with the target of the CRISPR/Cas9 system. Red line and letters, PAM sequence; green line and letters, sequence complementary to the gRNA; purple letters, nucleotides being part of the *URA*3 locus; blue letters, nucleotides upstream and downstream of the *URA*3 locus; pink, italic letters, inserted nucleotides; Strain, strains analyzed; WT, wild-type; strain numbers starting with 1, transformants obtained from the first transformation; strain numbers starting with 2, transformants obtained from the second transformation; Start, genomic position in the WT. Sequence, obtained sequencing result; End, genomic position in the WT; Del, number of nucleotides deleted

From the first transformation assay, two transformants resistant to hygromycin B and 5-FOA were investigated by colony PCR (using primers 7 and 8, see Supplementary Table 1) and subsequent sequencing. One transformant, named 1.1, had a deletion of 39 bp and insertion of one nucleotide (Fig. 2). For the other transformant, 1.2, neither colony PCR nor PCR on extracted genomic DNA succeeded with primers 7 and 8. Hence, a different set of primers (primers 9 and 10) were used, amplifying a longer sequence. A deletion of 862 bp was revealed and confirmed by sequencing (Fig. 2).

As the colony PCR did not work reliably, the transformants from the second transformation were verified with PCR on genomic DNA. After the PCRs, 21 out of 33 PCR products were selected based on their diversity of estimated size on the gel and sent for sequencing with primers 9, 10 or 11 (Supplementary Table 1). Deletions from 6 to 1187 bp were observed, and insertions were present in 3 transformants. The results of the sequencing are presented in Fig. 2.

### Verification of the loss of hygromycin B resistance

**Fig. 3:**
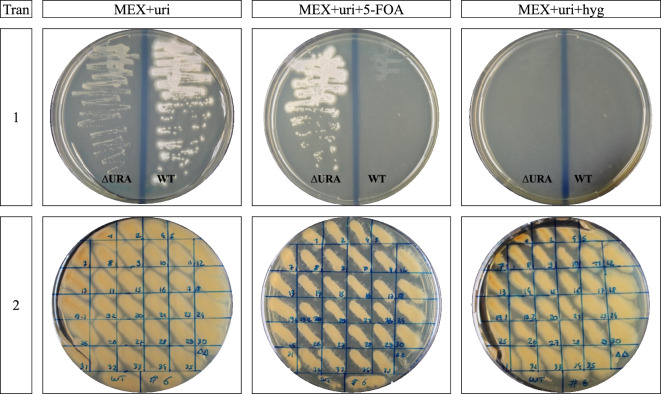
Phenotype of selected transformants. Growth of the wild-type and obtained transformants was tested on plates with the indicated medium. From the first transformation assay (row 1) the transformant 1.2 (∆URA) and the WT are displayed. From the second transformation (row 2) the 33 selected transformants were tested and are displayed together with the wild-type (WT), the URA3 mutant strain #6 from [19] (#6), and an A. pullulans EXF-150 double deletion mutant with a hygromycin B genomic resistance cassette (∆∆). *Tran* number of transformations, *MEX* malt extract, *uri* uridine, *hyg* hygromycin B, *5-FOA* 5-fluoroorotic acid

To test how quickly the *A. pullulans* transformants lose the CRISPR/Cas9 plasmid, transformants were cultivated on non-selective media before testing again their capacity to grow on media containing hygromycin B. One transformant from the first transformation, transformant 1.2, was randomly selected and after two passages of isolation on MEX plates with uridine. It grew on MEX plates with uridine, MEX plates supplemented with 5-FOA, but not on MEX plates supplemented with hygromycin B (Fig. 3). The 33 selected transformants from the second transformation were all still able to grow on MEX plates containing hygromycin B after two passages of isolation on MEX plates with uridine (Fig. 3). We can therefore not confirm that *A. pullulans* quickly loses the plasmid in a reproducible way.

## Discussion

The presented results demonstrate that deletions can be efficiently generated in *A. pullulans* using the AMA1 CRISPR/Cas9 plasmid-based system. 80–100% of the tested hygromycin-B resistant transformants also exhibited 5-FOA resistance, suggesting deletion of the *URA*3 function, confirmed by sequencing of the *URA*3 locus. The presented system is easier to handle and adapt than existing systems for *A. pullulans*. Only two primers need to be modified to adapt the plasmid to a target gene, the remaining elements are standard, and the plasmids are available at Addgene, ensuring transparency and reliability through sequencing and mapping. No step requires RNAse-free condition and the transformation can be performed with frozen protoplasts, enabling time-saving operations and standardization (many protoplasts can be generated at once and stored in aliquots). However, further testing is required to assess the impact of prolonged protoplast storage on transformation efficiency (we did not store the protoplasts over 6 months). The presented system is cost-effective, with in vivo Cas9 synthesis reducing expenses.

The loss of the hygromycin B resistance and the plasmid seemed random, occurring in the only extensively tested transformant of the first transformation, transformant 1.2, but not in the transformants of the second transformation. Further investigation is needed to understand this discrepancy and to optimize assay parameters for a rapid plasmid loss The latter ensures a transient Cas9 expression and the prevention of any off-target modifications and of the genomic integration of the hygromycin B resistance, which would hamper the possibility to recycle the marker for subsequent deletions.

The selection of the 20-nucleotide sequence, which defines the target DNA of Cas9, is a crucial step of this method. This sequence will influence the properties of two primers for the MOE-PCR. The primers should ideally have a GC-content between 40 and 60%, melting temperatures that differ less than 3 °C and avoid single nucleotide repeats or strong secondary structures. Additionally, this sequence should be close to a PAM sequence and be unique in the genome. Meeting these criteria can be challenging in some genome regions, such as GC rich regions.

A surprising observation was to obtain a mutant with an 862 bp deletion with a single gRNA. Large deletions can be advantageous to target a complete gene deletion but it can also cause unwanted deletions of neighbouring genes. This phenomenon was not reported in the other studies using the CRISPR/Cas9 method in *A. pullulans*. However, we suspect that the same phenomenon happened to Kreuter et al. [19]. They used two gRNAs to target *URA*3. Out of the six strains, four showed deletions of 6–29 bp at the site of the gRNA1 only, with no deletion at the site of gRNA2 [19]. For two strains, they observed 821–843 bp deletions between the gRNA1 site and the gRNA2 site [19]. Surprisingly, for both strains, the PAM sequence of the gRNA2 was unmodified [19]. Therefore, we assume that these large deletions were due to gRNA1 only and that gRNA2 did not lead to any cut in the DNA. The 20 bp nucleotide target sequence of the gRNA used in this study was the same as used by Kreuter et al. for the gRNA1 [19]. This phenomenon was not reported in the work from Zhang et al. [20]. Either it is specific for the gRNA used in this study and by Kreuter et al., or it could be specific to the strain EXF-150, also used by Kreuter et al. and in this work, while Zhang et al. used strain CCTCCM2012223 [19, 20]. Both strains are wild-types with a functional non-homologous end joining mechanism.

We also observed that the colony PCR method was sensitive to the amount of cell mass dissolved in NaOH although no quantity specifications were provided in the original protocol [30]. Therefore, we would recommend to optimize the OD solution suitable for the colony PCR for the particular strain to be transformed.

The used GGA system, was originally designed for pathway construction in *Aspergillus*, can be simplified for deletions by using only one GGA and modifying the standard primers for the gRNA fragment assembly [28]. The *Bsa*I cutting site can be replaced by a *Bbs*I allowing direct assembly of the gRNA fragment within the plasmid containing the Cas9 expression cassette.

The ability of *A. pullulans* to express the AMA1 CRISPR/Cas9 suggests that promoters and terminators from other fungi can be used in *A. pullulans* as already suggested by previous studies [20, 31]. The tested plasmid contains promoters and terminators from *A. niger* (p*coxA* controlling the Cas9 expression, p*mbfA* controlling the gRNA expression) and *T. reesei* (p*pki* controlling the hygromycin B resistance) Previously, the transcriptional start points in the promoter of the *gla*A gene was shown to be identical in *A. niger* and *A. pullulans*, suggesting this promoter could be used in both species [31] and, Zhang et al. demonstrated that promoters and terminators commonly used in yeast also function in *A. pullulans* [20]. Combining this knowledge with promoter and terminator characterization in *A. pullulans* could lead to a robust toolbox allowing the use of the AMA1 CRISPR/Cas9 plasmid for pathway engineering in *A. pullulans.*

## Conclusion

In this study, *A. pullulans* deletion strains were successfully created using an AMA1-based CRISPR/Cas9 single plasmid system. This system is easy to adapt to different targets and is the most accessible of the existing systems for *A. pullulans*. No high-cost material is required, and all necessary plasmids are in a repository. This system provides a new opportunity for constructing multiple deletion strains in *A. pullulans*. Further exploration is required to pinpoint parameters leading to early rejection of the plasmid and the consequent release from resistance, enhancing the potential of this system for generating marker-free deletions. Additional exploration could also validate the efficacy of the system in DNA insertions, enabling intensive engineering of *A. pullulans* and accelerating the understanding of this enigmatic fungus.

### Supplementary Information

Additional file 1: Figure 1. Phenotype of 127 transformants from the first transformation. Transformants from the first transformation, which were resistant to hygromycin B, were randomly selected and transferred on MEX plates supplemented with uridine and 5-FOA. Transformants were tested and are displayed together with the wild-type (WT) as negative control (-), and the *URA3* mutant strain #6 from [19] (#6) as positive control (+). Plates were incubated for 3 (left) or 4 (right) days at 24°C. MEX, malt extract; uri, uridine; 5-FOA, 5-fluoroorotic acid.

Additional file 2.

Additional file 3.

## Data Availability

This published article and its supplementary information files include all data generated or analyzed during this study. The plasmids used for the GGA are available at Addgene under the references 89915 and 90278 (see Supplementary Table 1).

## Abbreviations

AMA1: Autonomously maintained in *Aspergillus*
CFU: Colony-forming unit
HDV: Hepatititis delta virus
hyg: Hygromycin B
kanR: Kanamycin resistance cassette
OD_600_: Optical density at 600 nm
Ori: Origin of replication
PAM: Protospacer adjacent motif
PEG: Polyethylene glycol
gRNA: Guide RNA
*URA3*: Gene coding for orotidine 5-phosphate decarboxylase
WT: Wild-type *A. pullulans* EXF-150
5-FOA: 5-Fluoroorotic acid
