# Supplementary figures and images for: Genomic deletions in Aureobasidium pullulans by an AMA1 plasmid for gRNA and CRISPR/Cas9 expression
Source: Fungal Biol Biotechnol. 2024 Jun 1;11:6. doi: 10.1186/s40694-024-00175-4 (PMC11143684; doi:10.1186/s40694-024-00175-4)

# MEX+uri+5-FOA

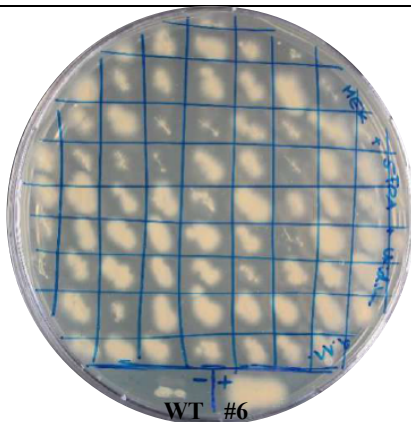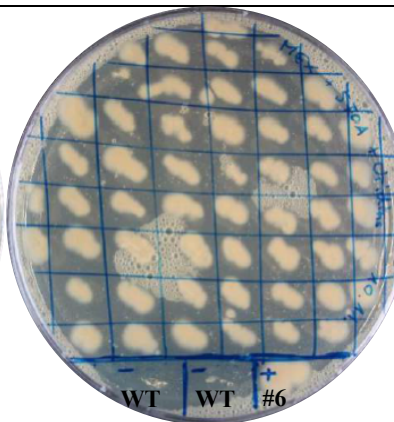

Supplement: Supplementary file 1 — Additional file 1: Figure 1. Phenotype of 127 transformants from the first transformation. Transformants from the first transformation, which were resistant to hygromycin B, were randomly selected and transferred on MEX plates supplemented with uridine and 5-FOA. Transformants were tested and are displayed together with the wild-type (WT) as negative control (-), and the URA3 mutant strain #6 from [19] (#6) as positive control (+). Plates were incubated for 3 (left) or 4 (right) days at 24°C. MEX, malt extract; uri, uridine; 5-FOA, 5-fluoroorotic acid. [file 40694_2024_175_MOESM1_ESM.pdf]
