# Supplementary material for: Genomic deletions in Aureobasidium pullulans by an AMA1 plasmid for gRNA and CRISPR/Cas9 expression
Source: Fungal Biol Biotechnol. 2024 Jun 1;11:6. doi: 10.1186/s40694-024-00175-4 (PMC11143684; doi:10.1186/s40694-024-00175-4)
Supplement: Supplementary file 2 — Additional file 2. [file 40694_2024_175_MOESM2_ESM.docx]

**Supplementary material**

**Supplementary Table 1: Oligonucleotides and plasmids used in this study.**

| N° | Name | Sequence (5´-3´) | Description |
| --- | --- | --- | --- |
| 1 | constructionprimer_1 | ATGGTCTCCCATGGCGGTCCTGATGAGTCCGTGAGGACGAAACGAGTAAGCTCGTC**GACC**^1^ | Primer for gRNA construction, primer adapted to the *URA*3 target |
| 2 | constructionprimer_2 | AAACGAGTAAGCTCGTC**GACCGCAAGTTTGGCGACAT**GTTTTAGAGCTAGAAATAGCAAG | Primer for gRNA construction, primer adapted to the *URA*3 target |
| 3 | 1gRNA_all_rev | CGCCATGCCGAAGCATGTTGCCCAGCCGGCGCCAGCGAGGAGGCTGGGACCATGCCGGCC | Primer for gRNA construction, generic primer [28] |
| 4 | 2gRNA_all_rev | TAGGTCTCCAAGCAGTCCAAAGCTGTCCCATTCGCCATGCCGAAGCATGTTGCCCAGCCG | Primer for gRNA construction, generic primer [28] |
| 5 | 3gRNA_all_rev | AGGCTGGGACCATGCCGGCCAAAAGCACCGACTCGGTGCCACTTTTTCAAGTTGATAACG | Primer for gRNA construction, generic primer [28] |
| 6 | 4gRNA_all_fw | GTTTTAGAGCTAGAAATAGCAAGTTAAAATAAGGCTAGTCCGTTATCAACTTGAAAAAGT | Primer for gRNA construction, generic primer [28] |
| 7 | Apul_*URA*3_seq2_fwd | CCTACGATTGATCGCAGC | Primer for *URA*3 locus PCR [19] |
| 8 | Apul_*URA*3_seq2_rev | GTAGCAGCGCCAATTCTC | Primer for *URA*3 locus PCR [19] |
| 9 | AP_*URA*3_3.1 kb_Fw | CAGATCCGCCTGAACCCTAC | Primer for *URA*3 locus PCR, for generation of a 3.1 kb fragment in WT with primer 10 |
| 10 | AP_*URA*3_3.1 kb_Rv | CTAGCAGGGCTGCGATAGAC | Primer for *URA*3 locus PCR, for generation of a 3.1 kb fragment in WT with primer 9 |
| 11 | Apul_*URA*3_rev | TCACCTGCTACCTTTTGTTGC | Primer for *URA*3 locus PCR |
| 12 | AP_*URA*3_3.7kb_Fw | TAACGAGAGGCTTTGGGCAG | Primer for *URA*3 locus PCR, for generation of a 3.7 kb fragment in WT with primer 13 |
| 13 | AP_*URA*3_3.7kb_Rv | ACCTTCTTCAAGGGCAGAGC | Primer for *URA*3 locus PCR, for generation of a 3.7 kb fragment in WT with primer 12 |
| 14 | Complementary sequence for gRNA targeting *URA*3 | GACCGCAAGTTTGGCGACAT | Target used in [19] and used here to generate the oligonucleotide for the gRNA fragment construction |
| P1 | BB11_L_23_syn_*Bsa*I | See Addgene | Plasmid for the first GGA, Addgene ref 89915, [28] |
| P2 | pMST665_BB3_L23_gRNA empty_cas9_BbsI | See Addgene | Plasmid for the second GGA, Addgene ref 90278, [28] |

^1^ red, bold letters indicate the 20-nucleotide sequence that defines the genomic target of the gRNA; in order to adapt the system to another genomic target, only the 20-nucleotide sequence needs to be adjusted.
