# Supplementary material for: Genomic deletions in Aureobasidium pullulans by an AMA1 plasmid for gRNA and CRISPR/Cas9 expression
Source: Fungal Biol Biotechnol. 2024 Jun 1;11:6. doi: 10.1186/s40694-024-00175-4 (PMC11143684; doi:10.1186/s40694-024-00175-4)
Supplement: Supplementary file 3 — Additional file 3. [file 40694_2024_175_MOESM3_ESM.docx]

**Supplementary sequences**

**Sequence 1. 248 bp fragment containing the sequence for gRNA expression**

Green letters, hammerhead ribozyme; red letters, the complementary sequence to the target DNA of the gRNA (20 nucleotide spacer); blue letters, the gRNA scaffold; and brown letters, the HDV ribozyme.

>atggtctcccatggcggtcctgatgagtccgtgaggacgaaacgagtaagctcgtcgaccgcaagtttggcgacatgttttagagctagaaatagcaagttaaaataaggctagtccgttatcaacttgaaaaagtggcaccgagtcggtgcttttggccggcatggtcccagcctcctcgctggcgccggctgggcaacatgcttcggcatggcgaatgggacagctttggactgcttggagaccta

**Sequence 2. CRISPR/Cas9 plasmid (14,715 bp) generated in this study**

Blue letters, the gRNA sequence; purple letters, the Cas9 sequence; red letters, the Kozak sequence; green letters, the hygromycin B resistance sequence; orange letters, the origin of the replication sequence.

>gcttgatccacttaacgttactgaaatcatcaaacagcttgacgaatctggatataagatcgttggtgtcgatgtcagctccggagttgagacaaatggtgttcaggatctcgataagatacgttcatttgtccaagcagcaaagagtgccttctagtgatttaatagctccatgtcaacaagaataaaacgcgtttcgggtttacctcttccagatacagctcatctgcaatgcattaatgcattggacctcgcaaccctagtacgcccttcaggctccggcgaagcagaagaatagcttagcagagtctattttcattttcgggagacgagatcaagcagatcaacggtcgtcaagagtcctacgagactgaggaatccgctcttggctccacgcgactatatatttgtctctaattgtactttgacatgctcctcttctttactctgatagcttgactatgaaaattccgtcaccagcccctgggttcgcaaagataattgcactgtttcttccttgaactctcaagcctacaggacacacattcatcgtaggtataaacctcgaaaatcattcctactaagatgggtatacaatagtaaccatgcatggttgcctagtgaatgctccgtaacacccaatacgccggccgaaacttttttacaactctcctatgagtcgtttacccagaatgcacaggtacacttgtttagaggtaatccttctttctagaagtcctcgtgtactgtgtaagcgcccactccacatctccactcgctccggggagaaccgaagaggatgggaaccccgaccatggcctgcggccgcagctatcagtcctctcagctgacgcccctcacgaaccctcgcactctcccgaaatgctcttcggtgtccatggcgcatcatctcagcagcatcccatgtcaaaccatggctttgggcccacggactctgtggccctgccgcaacatcaccatcaccatcgactcccgccccatgcagcgctgcgcgccccagggcgtcatggagtgaatgtggaatctcctcctttgccctcgggcccgccgagtgtggtgggccagcctggaatgcctgatccagcccccaggcctcgaggaccaaaactgaagtttactcccgaagaggacgctctactggtggagttgaaggaaaacaagaacttgacgtggaagcaaattgcagacttcttcccgggccgaacgagcggtaccttgcaagtccgatactgcaccaagctgaaggctaaggatgtagcttggagtgacgaaatggttcgatttgctcctgatgtatttctacgcctgtctcacacatgctaatgaagggaataggtacaaaggctgcagcgggcaatgcacgagtacgagaacgatcggtggcgcatcattgcagggaaggttggaaatggcttcaccccagctgcttgccgcgagaaagccatgcagctccatgagtaaaagcgttggggaattttcatatttatatctactgtcgccagattcggccctgcttggaccctctgatctccttactctccatattggttcaaatgtcgggtcaccgatagggctggtggtgcaggcttgttgtaggcacgggaggatgatcagcataactctgagtcactatagggacgggttgatgtaaggtattaagtgatgtatgataattcattttagcccgggggaacatatggcgccggcatttgttcgttcgcaatgaaccgacactagcgtccgctctcgcagtttagcaccggctgatcccgggctgaacgcggccattgctcggccggggcatgtgttccttatctacggcagaccgcagatgaccactggagcagattatagaccctaagccctaagccggacacccaatcgagtaggtctgcggaccaggtcactgcgggcagccggagaagctccgcaaccaatcaatccccggcgctgactaagggcaggcgaccacgggccgaagcggcttcaaactcacctcaacctccaaactccctcatctccaaacgtccttgccttgtctgccgtcattgcaacccacccaccaggacacatggacaagaagtactccattgggctcgatatcggcacaaacagcgtcggttgggccgtcattacggacgagtacaaggtgccgagcaaaaaattcaaagttctgggcaataccgatcgccacagcataaagaagaacctcattggcgccctcctgttcgactccggggagacggccgaagccacgcggctcaaaagaacagcacggcgcagatatacccgcagaaagaatcggatctgctacctgcaggagatctttagtaatgagatggctaaggtggatgactctttcttccataggctggaggagtcctttttggtggaggaggataaaaagcacgagcgccacccaatctttggcaatatcgtggacgaggtggcgtaccatgaaaagtacccaaccatatatcatctgaggaagaagcttgtagacagtactgataaggctgacttgcggttgatctatctcgcgctggcgcatatgatcaaatttcggggacacttcctcatcgagggggacctgaacccagacaacagcgatgtcgacaaactctttatccaactggttcagacttacaatcagcttttcgaagagaacccgatcaacgcatccggagttgacgccaaagcaatcctgagcgctaggctgtccaaatcccggcggctcgaaaacctcatcgcacagctccctggggagaagaagaacggcctgtttggtaatcttatcgccctgtcactcgggctgacccccaactttaaatctaacttcgacctggccgaagatgccaagcttcaactgagcaaagacacctacgatgatgatctcgacaatctgctggcccagatcggcgaccagtacgcagacctttttttggcggcaaagaacctgtcagacgccattctgctgagtgatattctgcgagtgaacacggagatcaccaaagctccgctgagcgctagtatgatcaagcgctatgatgagcaccaccaagacttgactttgctgaaggcccttgtcagacagcaactgcctgagaagtacaaggaaattttcttcgatcagtctaaaaatggctacgccggatacattgacggcggagcaagccaggaggaattttacaaatttattaagcccatcttggaaaaaatggacggcaccgaggagctgctggtaaagcttaacagagaagatctgttgcgcaaacagcgcactttcgacaatggaagcatcccccaccagattcacctgggcgaactgcacgctatcctcaggcggcaagaggatttctacccctttttgaaagataacagggaaaagattgagaaaatcctcacatttcggataccctactatgtaggccccctcgcccggggaaattccagattcgcgtggatgactcgcaaatcagaagagactatcactccctggaacttcgaggaagtcgtggataagggggcctctgcccagtccttcatcgaaaggatgactaactttgataaaaatctgcctaacgaaaaggtgcttcctaaacactctctgctgtacgagtacttcacagtttataacgagctcaccaaggtcaaatacgtcacagaagggatgagaaagccagcattcctgtctggagagcagaagaaagctatcgtggacctcctcttcaagacgaaccggaaagttaccgtgaaacagctcaaagaggactatttcaaaaagattgaatgtttcgactctgttgaaatcagcggagtggaggatcgcttcaacgcatccctgggaacgtatcacgatctcctgaaaatcattaaagacaaggacttcctggacaatgaggagaacgaggacattcttgaggacattgtcctcacccttacgttgtttgaagatagggagatgattgaagaacgcttgaaaacttacgctcatctcttcgacgacaaagtcatgaaacagctcaagaggcgccgatatacaggatgggggcggctgtcaagaaaactgatcaatgggatccgagacaagcagagtggaaagacaatcctggattttcttaagtccgatggatttgccaaccggaacttcatgcagttgatccatgatgactctctcacctttaaggaggacatccagaaagcacaagtttctggccagggggacagccttcacgagcacatcgctaatcttgcaggtagcccagctatcaaaaagggaatactgcagaccgttaaggtcgtggatgaactcgtcaaagtaatgggaaggcataagcccgagaatatcgttatcgagatggcccgagagaaccaaactacccagaagggacagaagaacagtagggaaaggatgaagaggattgaagagggtataaaagaactggggtcccaaatccttaaggaacacccagttgaaaacacccagcttcagaatgagaagctctacctgtactacctgcagaacggcagggacatgtacgtggatcaggaactggacatcaatcggctctccgactacgacgtggatcatatcgtgccccagtcttttctcaaagatgattctattgataataaagtgttgacaagatccgataaaaatagagggaagagtgataacgtcccctcagaagaagttgtcaagaaaatgaaaaattattggcggcagctgctgaacgccaaactgatcacacaacggaagttcgataatctgactaaggctgaacgaggtggcctgtctgagttggataaagccggcttcatcaaaaggcagcttgttgagacacgccagatcaccaagcacgtggcccaaattctcgattcacgcatgaacaccaagtacgatgaaaatgacaaactgattcgagaggtgaaagttattactctgaagtctaagctcgtctcagatttcagaaaggactttcagttttataaggtgagagagatcaacaattaccaccatgcgcatgatgcctacctgaatgcagtggtaggcactgcacttatcaaaaaatatcccaagcttgaatctgaatttgtttacggagactataaagtgtacgatgttaggaaaatgatcgcaaagtctgagcaggaaataggcaaggccaccgctaagtacttcttttacagcaatattatgaattttttcaagaccgagattacactggccaatggagagattcggaagcgaccacttatcgaaacaaacggagaaacaggagaaatcgtgtgggacaagggtagggatttcgcgacagtccggaaggtcctgtccatgccgcaggtgaacatcgttaaaaagaccgaagtacagaccggaggcttctccaaggaaagtatcctcccgaaaaggaacagcgacaagctgatcgcacgcaaaaaagattgggaccccaagaaatacggcggattcgattctcctacagtcgcttacagtgtactggttgtggccaaagtggagaaagggaagtctaaaaaactcaaaagcgtcaaggaactgctgggcatcacaatcatggagcgatcaagcttcgaaaaaaaccccatcgactttctcgaggcgaaaggatataaagaggtcaaaaaagacctcatcattaagcttcccaagtactctctctttgagcttgaaaacggccggaaacgaatgctcgctagtgcgggcgagctgcagaaaggtaacgagctggcactgccctctaaatacgttaatttcttgtatctggccagccactatgaaaagctcaaagggtcccccgaagataatgagcagaagcagctgttcgtggaacaacacaaacactaccttgatgagatcatcgagcaaataagcgaattctccaaaagagtgatcctcgccgacgctaacctcgataaggtgctttctgcttacaataagcacagggataagcccatcagggagcaggcagaaaacattatccacttgtttactctgaccaacttgggcgcgcctgcagccttcaagtacttcgacaccaccatagacagaaagcggtacacctctacaaaggaggtcctggacgccacactgattcatcagtcaattacggggctctatgaaacaagaatcgacctctctcagctcggtggagacagcagggctgaccccaagaagaagaggaaggtgtgagcttgatccacttaacgttactgaaatcatcaaacagcttgacgaatctggatataagatcgttggtgtcgatgtcagctccggagttgagacaaatggtgttcaggatctcgataagatacgttcatttgtccaagcagcaaagagtgccttctagtgatttaatagctccatgtcaacaagaataaaacgcgtttcgggtttacctcttccagatacagctcatctgcaatgcattaatgcattggacctcgcaaccctagtacgcccttcaggctccggcgaagcagaagaatagcttagcagagtctattttcattttcgggagacgagatcaagcagatcaacggtcgtcaagagtcctacgagactgaggaatccgctcttggctccacgcgactatatatttgtctctaattgtactttgacatgctcctcttctttactctgatagcttgactatgaaaattccgtcaccagcccctgggttcgcaaagataattgcactgtttcttccttgaactctcaagcctacaggacacacattcatcgtaggtataaacctcgaaaatcattcctactaagatgggtatacaatagtaaccatgcatggttgcctagtgaatgctccgtaacacccaatacgccggccgaaacttttttacaactctcctatgagtcgtttacccagaatgcacaggtacacttgtttagaggtaatccttctttctagaagtcctcgtgtactgtgtaagcgcccactccacatctccactcgctaattcatgaataacggtgagactagcggccggtccccttatcccagctgttccacgttggcctgcccctcagttagcgctcaactcaatgcccctcactggcgaggcgagggcaaggatggaggggcagcatcgcctgagttggagcaaagcggccgccatgggagcagcgaaccaacggagggatgccgtgctttgtcgtggctgctgtggccaatccgggcccttggttggctcacagagcgttgctgtgagtccatgagctattattgctaggtacagtatagagagaggagagagagagagagagagagaggggaaaaaaggtgaggttgaagtgagaaaaaaaaaaaaaaaaatccaaccactgacggctgccggctctgccacccccctccctccaccccagaccacctgcacactcagcgcgcagcatcacctaatcttggctcgccttcccgcagctcaggttgttttttttttctctctccctcgtcgaagccgcccttgttcccttatttatttccctctccatccttgtctgcctttggtccatctgcccctttgtctgcatctcttttgcacgcatcgccttatcgtcgtctcttttttcactcacgggagcttgacgatgacctgactcgtgagcctcacctgctgatttctctccccccctcccgaccggcttgacttttgtttctcctccagtaccttatcgcgaagccggaagaacctcttaacctctagatgaaaaagcctgaactcaccgcgacgtctgtcgagaagttcctgatcgaaaagttcgacagcgtctccgacctgatgcagctctcggagggcgaagaatctcgtgctttcagcttcgatgtaggagggcgtggatatgtcctgcgggtaaatagctgcgccgatggtttctacaaagatcgttatgtttatcggcactttgcatcggccgcgctcccgattccggaagtgcttgacattggggaattcagcgagagcctgacctattgcatctcccgccgtgcacagggtgtcacgttgcaagacctgcctgaaaccgaactgcccgctgttctgcagccggtcgcggaggccatggatgcgatcgctgcggccgatctcagccagacgagcgggttcggcccattcggaccgcaaggaatcggtcaatacactacatggcgtgatttcatatgcgcgattgctgatccccatgtgtatcactggcaaactgtgatggacgacaccgtcagtgcgtccgtcgcgcaggctctcgatgagctgatgctttgggccgaggactgccccgaagtccggcacctcgtgcacgcggatttcggctccaacaatgtcctgacggacaatggccgcataacagcggtcattgactggagcgaggcgatgttcggggattcccaatacgaggtcgccaacatcttcttctggaggccgtggttggcttgtatggagcagcagacgcgctacttcgagcggaggcacccggagcttgcaggatcgccgcggctccgggcgtatatgctccgcattggtcttgaccaactctatcagagcttggttgacggcaatttcgatgatgcagcttgggcgcagggtcgatgcgacgcaatcgtccgatccggagccgggactgtcgggcgtacacaaatcgcccgcagaagcgcggccgtctggaccgatggctgtgtagaagtactcgccgatagtggaaaccgacgccccagcactcgtccgagggcaaaggaatagatgcatggctttcgtgaccgggcttcaaacaatgatgtgcgatggtgtggttcccggttggcggagtctttgtctactttggttgtctgtcgcaggtcggtagaccgcaaatgagcaactgatggattgttgccagcgatactataattcacatggatggtctttgtcgatcagtagctagtgagagagagagaacatctatccacaatgtcgagtgtctattagacatactccgagaataaagtcaactgtgtctgtgatctaaagatcgattcggcagtcgagtagcgtataacaactccgagtaccagcgaaagcacgtcgtgacaggagcagggctttgccaactgcgcaaccttgcttgaatgaggatacacggggtgcaacatggctgtactgatccatcgcaaccaaaatttctgtttatagatcaagctggtagattccaattactccacctcttgcgcttctccatgacatgtaagtgcacgtggaaaccatacccaaattgcctacagctgcggagcatgagcctatggcgatcagtctggtcatgttaaccagcctgtgctctgacgttaatgcagaatagcttatgctgcagacgcgttacgtatcggatccagaattcgtgatatctgaattcgtcgacaagcttattttttgtatactgttttgtgatagcacgaagtttttccacggtatcttgttaaaaatatatatttgtggcgggcttacctacatcaaattaataagagactaattataaactaaacacacaagcaagctactttagggtaaaagtttataaatgcttttgacgtataaacgttgcttgtatttattattacaattaaaggtggatagaaaacctagagactagttagaaactaatctcaggtttgcgttaaactaaatcagagcccgagaggttaacagaacctagaaggggactagatatccgggtagggaaacaaaaaaaaaaaacaagacagccacatattagggagactagttagaagctagttccaggactaggaaaataaaagacaatgataccacagtctagttgacaactagatagattctagattgaggccaaagtctctgagatccaggttagttgcaactaatactagttagtatctagtctcctataactctgaagctagaataacttactactattatcctcaccactgttcagctgcgcaaacggagtgattgcaaggtgttcagagactagttattgactagtcagtgactagcaataactaacaaggtattaacctaccatgtctgccatcaccctgcacttcctcgggctcagcagccttttcctcctcattttcatgctcattttccttgtttaagactgtgactagtcaaagactagtccagaaccacaaaggagaaatgtcttaccactttcttcattgcttgtctcttttgcattatccatgtctgcaactagttagagtctagttagtgactagtccgacgaggacttgcttgtctccggattgttggaggaactctccagggcctcaagatccacaacagagccttctagatgactggtcaataactagttggtctttgtctgagtctgactgacttacgaggttgcatactcgctccctttgcctcgtcaatcgatgagaaaaagcgccaaaactcgcaatatggctttgaaccacacggtgctgagactagttagaatctagtcccaaactagcttggatagcttacctttgccctttgcgttgcgacaggtcttgcagggtatggttcctttctcaccagctgatttagctgccttgctaccctcacggcggatctgcataaagagtggctagaggttataaattagcactgatcctaggtacggggctgaatgtaacttgcctttcctttctcatcgcgcggcaagacaggcttgctcaaattcctaccagtcacaggggtatgcacggcgtacggaccacttgaactagtcacagattagttagcaactagtctgcattgaatggctgtacttacgggccctcgccattgtcctgatcatttccagcttcaccctcgttgctgcaaagtagttagtgactagtcaaggactagttgaaatgggagaagaaactcacgaattctcgacacccttagtattgtggtccttggacttggtgctgctatatattagctaatacactagttagactcacagaaacttacgcagctcgcttgcgcttcttggtaggagtcggggttgggagaacagtgccttcaaacaagccttcataccatgctacttgactagtcagggactagtcaccaagtaatctagataggacttgcctttggcctccatcagttccttcatagtgggaggtccattgtgcaatgtaaactccatgccgtgggagttcttgtccttcaagtgcttgaccaatatgtttctgttggcagagggaacctgtcaactagttaataactagtcagaaactagtatagcagtagactcactgtacgcttgaggcatcccttcactcggcagtagacttcatatggatggatatcaggcacgccattgtcgtcctgtggactagtcagtaactaggcttaaagctagtcgggtcggcttactatcttgaaatccggcagcgtaagctccccgtccttaactgcctcgagatagtgacagtactctggggactttcggagatcgttatcgcgaatgctcggcatactaatcgttgactagtcttggactagtcccgagcaaaaaggattggaggaggaggaggaaggtgagagtgagacaaagagcgaaataagagcttcaaaggctatctctaagcagtatgaaggttaagtatctagttcttgactagatttaaaagagatttcgactagttatgtacctggagtttggatataggaatgtgttgtggtaacgaaatgtaagggggaggaaagaaaaagtcggtcaagaggtaactctaagtcggccattcctttttgggaggcgctaaccataaacggcatggtcgacttagagttagctcagggaatttagggagttatctgcgaccaccgaggaacggcggaatgccaaagaatcccgatggagctctagctggcggttgacaaccccaccttttggcgtttctgcggcgttgcaggcgggactggatacttcgtagaaccagaaaggcaaggcagaacgcgctcagcaagagtgttggaagtgatagcatgatgtgccttgttaactaggtcaaaatctgcagtatgcttgatgttatccaaagtgtgagagaggaaggtccaaacatacacgattgggagagggcctaggtataagagtttttgagtagaacgcatgtgagcccagccatctcgaggagattaaacacgggccggcatttgatggctatgttagtaccccaatggaaagcctgagagtccagtggcgctgtgagcaaaaggccagcaaaaggccaggaaccgtaaaaaggccgcgttgctggcgtttttccataggctccgcccccctgacgagcatcacaaaaatcgacgctcaagtcagaggtggcgaaacccgacaggactataaagataccaggcgtttccccctggaagctccctcgtgcgctctcctgttccgaccctgccgcttaccggatacctgtccgcctttctcccttcgggaagcgtggcgctttctcaatgctcacgctgtaggtatctcagttcggtgtaggtcgttcgctccaagctgggctgtgtgcacgaaccccccgttcagcccgaccgctgcgccttatccggtaactatcgtcttgagtccaacccggtaagacacgacttatcgccactggcagcagccactggtaacaggattagcagagcgaggtatgtaggcggtgctacagagttcttgaagtggtggcctaactacggctacactagaaggacagtatttggtatctgcgctctgctgaagccagttaccttcggaaaaagagttggtagctcttgatccggcaaacaaaccaccgctggtagcggtggtttttttgtttgcaagcagcagattacgcgcagaaaaaaaggatctcaagaagatcctttgatcttttctacggggtggagcgcaaaaaaccccgcccctgacagggcggggttttttcgcgatcggaggtacagtggccatgaaatccaatcatttccttctggccgccctcgggcaagagatagtgccgcagaggactctcacagcatctacatctgcgaccgcaacagccaccaagcgaggcgcacatgagcttgtcctcctcccatgccaaagtttggccctcttcgtttctgtgatgctgaaggaagtcaaactcgtcgatgataggaccagatggtttgtcaagggtcaacgctttccatgccttctggcaccggtagtaatgctcttctgcaagggagacttgacgtttcggatccgcgggccccgggacatgctggaagggattttctggctcaataccacgtctgtatttgaccctttccagacagttaatccgctgcaggagggcgaactgtagctcctcgttctccttgtagcgcttgatccagtctttttggatgttgcacttgcttggcctatgcttctcatataatcttgccctgtcatagagacgacgtctgagattgtagcgttcgtctttgatcacccggagccagataggcctgagtatatctgacattagatcaaagggtctgtggatagtctccttcagcatcagcgacgcatgtgactcgcatgtcggagagagcttgtgggtggtcatctttgatggcgtcctctgctttcccttgattttcgttgattgtttttcgaaagttaagtctggaagtcaagagaatccttctgccagacattatatttacgtatactgacgtagtagaaacagcgtcaggatgaggacatggtgtgtgctggaccacggaatcatagttcatcagtatattgggttggacaaataacgctgagcatgtatatgtctttacacactataaaagccagcgaacgccaataaaatagggcatattgatgtgaaaatatgacaccagttaaaagcagtgtattgattttatctctcttcacctcggacctatactaccgtatacaagactcaacttacttccagatatagtaatatacaccctatggacgaaccagcacaataattacagccaaacaacaccacccaaatggcatattcctaatcagcactaagcacaaataccactgtcatcacagcataatcaataagaatcccagacaaccgactcactctgactcaccttacacaaacccccaagcaaagcgcagcccagaacctcagccaacaatcgggcaacgtacggggaaagattggccgatccatgatgtcagcagccctaacccaaagcggactagcgcataccgcccctctgactccgccatcccagggctcgagaagcttccgtggcgtcgatataaattcagcgggccttgaacatccctccttacgacacacctcacgcgatcgattttgacactcacacaccgccaccctcacatcctccacccacaccacaccccttaatcaacccaccatcaccgctagaacgtctatctcatcaccgacttctcatccatcttcaaacatggcggtcctgatgagtccgtgaggacgaaacgagtaagctcgtcgaccgcaagtttggcgacatgttttagagctagaaatagcaagttaaaataaggctagtccgttatcaacttgaaaaagtggcaccgagtcggtgcttttggccggcatggtcccagcctcctcgctggcgccggctgggcaacatgcttcggcatggcgaatgggacagctttggact
